# Hydrate of Chloral in Singultus

**Published:** 1871-05

**Authors:** T. L. Leavitt

**Affiliations:** Germantown, Pa.


*Hydrate of Chloral in Singultus.* By T. L. Leavitt, M.D., of Germantown, Pa.

[From American Journal of the Medical Sciences.]

No new remedy has perhaps received such universal attention and trial as the hydrate of chloral, and justly so; for, during the brief period of its therapeutic existence, it has proved wonderfully successful in the very many instances of its exhibition. Numerous are the testimonies of practitioners as to its merits; insomnia, acute suffering, chronic diseases; and even child-bearing, have all been recorded as relieved through its potent agency.

To add one more to the extensive list of sufferings relieved as yet unannounced, I record the following cases of distressing singultus, which the physician has so frequently to contend with in cases of low fever and the general failing of vital power. Wearing and exhausting to the patient, and distressing to anxious friends, any pharmaceutical agent affording even temporary relief is hailed with eagerness, and most especially so after the wellworn changes have been rung unsuccessfully on musk, ether, the bromides, and the antispasmodics in general.

Mr. H., æt 60, after suffering for many years with an obscure disease, probably having its origin in the spinal cord, finally became so emaciated and debilitated as to be confined to his bed, where, after the first few days of gradual decline, a distressing and obstinate hiccough set in, producing rapid loss of strength and proving of great annoyance to the patient. Sulphuric ether in capsules, bromide of potassium and ammonium in solution, musk, camphor, &c., were all exhibited in turn, but failed to give any permanent relief, for they lost their controlling power after a few trials. The chloral hydrate was then used in five-grain doses in solution, and arrested almost immediately the singultus, and never afterwards failed to control the spasm in a most satisfactory manner, proving of the greatest comfort to the last remaining hours of the sick man.

Two other cases of obstinate hiccough, occurring subsequently in typhoid fever, were treated in the same manner, with the same pleasing result, the patients recovering finally.

It remains, of course, for more extended trial to fully establish this drug among the remedial agents in singultus, but certainly the results in the few cases coming under my notice have proved it to be of the highest value.

